# Effect of repetitive transcranial magnetic stimulation on chronobiological hypothalamic-pituitary-thyroid axis activity in major depression

**DOI:** 10.1192/j.eurpsy.2023.573

**Published:** 2023-07-19

**Authors:** F. Duval, M. C. Mokrani, V. Danila, A. Erb, F. Gonzalez Lopera, M. Tomsa

**Affiliations:** Pole 8/9 - Psychiatry, Centre Hospitalier, Rouffach, France

## Abstract

**Introduction:**

We previously demonstrated that the difference between 11 PM and 8 AM TSH response to protirelin (TRH) tests on the same day (∆∆TSH test) is an improved measure in detecting hypothalamic-pituitary-thyroid (HPT) axis dysregulation in depression. This chronobiological index is normalized after successful antidepressant treatment.

**Objectives:**

The present study aimed at assessing the effects of repetitive transcranial magnetic stimulation (rTMS) of the left dorsolateral prefrontal cortex (DLPFC) on the HPT axis activity in treatment resistant depressed inpatients (TRDs) (defined as having at least 2 treatment failures).

**Methods:**

The ∆∆TSH test was performed in 13 TRDs and 14 healthy hospitalized control subjects (HCs). To be enrolled in this study, patients had to show reduced ∆∆TSH values (i.e., < 2.5 mU/L) at baseline (BL). After 20 sessions of rTMS (using daily theta-burst stimulation; 100% resting motor threshold; number of pulses/session: 900), the ∆∆TSH test was repeated in all inpatients. *The* 17-item Hamilton depression rating scale *(HAM-D) was used to assess the severity of depression.* Remission was defined by a final HAM-D score ≤ 8.

**Results:**

Compared to BL, HAM-D scores decreased and ∆∆ TSH values increased after 20 sessions of rTMS (both p< 0.05 by T-test). There was a relationship between the reduction in HAM-D scores from BL to endpoint and the increase in ∆∆TSH values (rho = - 0.64; n = 13; p = 0.018). At endpoint, 7 patients showed ∆∆TSH normalization (among them 6 were remitters), while 6 patients did not normalize their ∆∆TSH (all were non-remitters) (p < 0.005 by Fisher Exact test).

**Conclusions:**

Our results suggest that after 20 sessions of rTMS, chronobiological restoration of the HPT axis activity is associated with clinical remission. Further investigation of the specific effects of rTMS on the HPT axis activity in TRDs is warranted.

**Disclosure of Interest:**

None Declared

